# Supplementary material for: Trends in Antimicrobial Resistance of Canine Otitis Pathogens in the Iberian Peninsula (2010–2021)
Source: Antibiotics (Basel). 2025 Mar 21;14(4):328. doi: 10.3390/antibiotics14040328 (PMC12024130; doi:10.3390/antibiotics14040328)
Supplement: Supplementary file 1 [file antibiotics-14-00328-s001.zip › antibiotics-3496522-supplementary.pdf]

**DRUG NAME**	**Organism/Organism Group**	**CLSI <= S**	**CLSI = I/SDD**	**CLSI >= R**
Amikacin	*Acinetobacter* species	16	32	64
Cefotaxime	*Acinetobacter* species	8	16-32	64
Ceftazidime	*Acinetobacter* species	8	16	32
Ciprofloxacin	*Acinetobacter* species	1	2	4
Doxycycline	*Acinetobacter* species	4	8	16
Gentamicin	*Acinetobacter* species	4	8	16
Minocycline	*Acinetobacter* species	4	8	16
Polymyxin B	*Acinetobacter* species	-	2	4
Tetracycline	*Acinetobacter* species	4	8	16
Tobramycin	*Acinetobacter* species	4	8	16
Trimethoprim-Sulfamethoxazole	*Acinetobacter* species	2/38	-	4/76
Amoxicillin-clavulanate	Anaerobes	4/2	8/4	16/8
Ampicillin	Anaerobes	0.5	1	2
Cefotaxime	Anaerobes	16	32	64
Cefoxitin	Anaerobes	16	32	64
Chloramphenicol	Anaerobes	8	16	32
Penicillin	Anaerobes	0.5	1	2
Tetracycline	Anaerobes	4	8	16
Ampicillin	Beta-hemolytic *Streptococcus*	0.25	-	-
Azithromycin	Beta-hemolytic *Streptococcus*	0.5	1	2
Cefotaxime	Beta-hemolytic *Streptococcus*	0.5	-	-
Chloramphenicol	Beta-hemolytic *Streptococcus*	4	8	16
Ciprofloxacin	Beta-hemolytic *Streptococcus*	-	-	-
Erythromycin	Beta-hemolytic *Streptococcus*	0.25	0.5	1
Penicillin	Beta-hemolytic *Streptococcus*	0.125	-	-
Tetracycline	Beta-hemolytic *Streptococcus*	2	4	8
Ceftazidime	*Burkholderia cepacia* complex	8	16	32
Chloramphenicol	*Burkholderia cepacia* complex	8	16	32
Minocycline	*Burkholderia cepacia* complex	4	8	16
Trimethoprim-Sulfamethoxazole	*Burkholderia cepacia* complex	2/38	-	4/76
Amikacin	Enterobacterales	4	8	16
Amoxicillin-Clavulanate	Enterobacterales	8/4	16/8	32/16
Ampicillin	Enterobacterales	8	16	32
Cefotaxime	Enterobacterales	1	2	4
Cefoxitin	Enterobacterales	8	16	32
Cefpodoxime	Enterobacterales	2	4	8
Ceftazidime	Enterobacterales	4	8	16
Chloramphenicol	Enterobacterales	8	16	32
Doxycycline	Enterobacterales	4	8	16
Gentamicin	Enterobacterales	2	-	8

Minocycline	Enterobacterales	4	8	16
Polymyxin B	Enterobacterales	-	2	4
Sulfamethoxazole	Enterobacterales	256		512
Tetracycline	Enterobacterales	4	8	16
Tobramycin	Enterobacterales	2	-	8
Trimethoprim-Sulfamethoxazole	Enterobacterales	2/38	-	4/76
Ciprofloxacin	Enterobacterales except *Salmonella* species	0.25	0.5	1
Azithromycin	Enterobacterales: *Salmonella enterica* ser. Typhi only	16	-	32
Ciprofloxacin	Enterobacterales: *Salmonella* species only	0.0625	0.12-0.5	1
Azithromycin	Enterobacterales: *Shigella* species only	8	16	32
Ampicillin	*Enterococcus* species	8	-	16
Chloramphenicol	*Enterococcus* species	8	16	32
Ciprofloxacin	*Enterococcus* species	1	2	4
Doxycycline	*Enterococcus* species	4	8	16
Erythromycin	*Enterococcus* species	0.5	1-4	8
Minocycline	*Enterococcus* species	4	8	16
Penicillin	*Enterococcus* species	8	-	16
Tetracycline	*Enterococcus* species	4	8	16
Amoxicillin-clavulanate	*Haemophilus influenzae* and *H. parainfluenzae*	2/1	4/2	8/4
Ampicillin	*Haemophilus influenzae* and *H. parainfluenzae*	1	2	4
Azithromycin	*Haemophilus influenzae* and *H. parainfluenzae*	4	-	-
Cefotaxime	*Haemophilus influenzae* and *H. parainfluenzae*	2	-	-
Cefpodoxime	*Haemophilus influenzae* and *H. parainfluenzae*	2	-	-
Ceftazidime	*Haemophilus influenzae* and *H. parainfluenzae*	2	-	-
Cefuroxime	*Haemophilus influenzae* and *H. parainfluenzae*	4	8	16
Cefuroxime	*Haemophilus influenzae* and *H. parainfluenzae*	4	8	16
Chloramphenicol	*Haemophilus influenzae* and *H. parainfluenzae*	2	4	8
Ciprofloxacin	*Haemophilus influenzae* and *H. parainfluenzae*	1	-	-
Tetracycline	*Haemophilus influenzae* and *H. parainfluenzae*	2	4	8
Trimethoprim-Sulfamethoxazole	*Haemophilus influenzae* and *H. parainfluenzae*	0.5/9.5	1/19-2/38	4/76
Azithromycin	*Neisseria gonorrhoeae*	1	-	-
Cefotaxime	*Neisseria gonorrhoeae*	0.5	-	-
Cefoxitin	*Neisseria gonorrhoeae*	2	4	8

Cefpodoxime	*Neisseria gonorrhoeae*	0.5	-	-
Ciprofloxacin	*Neisseria gonorrhoeae*	0.06	0.12-0.5	1
Penicillin	*Neisseria gonorrhoeae*	0.06	0.12-1	2
Tetracycline	*Neisseria gonorrhoeae*	0.25	0.5-1	2
Ampicillin	*Neisseria meningitidis*	0.12	0.25-1	2
Azithromycin	*Neisseria meningitidis*	2	-	-
Cefotaxime	*Neisseria meningitidis*	0.12	-	-
Chloramphenicol	*Neisseria meningitidis*	2	4	8
Ciprofloxacin	*Neisseria meningitidis*	0.03	0.06	0.12
Minocycline	*Neisseria meningitidis*	2	-	-
Penicillin	*Neisseria meningitidis*	0.06	0.12-0.25	0.5
Trimethoprim-Sulfamethoxazole	*Neisseria meningitidis*	0.12/2.4	0.25/4.75	0.5/9.5
Amikacin	Other Non-Enterobacterales	16	32	64
Cefotaxime	Other Non-Enterobacterales	8	16-32	64
Ceftazidime	Other Non-Enterobacterales	8	16	32
Chloramphenicol	Other Non-Enterobacterales	8	16	32
Ciprofloxacin	Other Non-Enterobacterales	1	2	4
Doxycycline	Other Non-Enterobacterales	4	8	16
Gentamicin	Other Non-Enterobacterales	4	8	16
Minocycline	Other Non-Enterobacterales	4	8	16
Tetracycline	Other Non-Enterobacterales	4	8	16
Tobramycin	Other Non-Enterobacterales	4	8	16
Trimethoprim-Sulfamethoxazole	Other Non-Enterobacterales	2/38	-	4/76
Amikacin	*Pseudomonas aeruginosa*	16	32	64
Ceftazidime	*Pseudomonas aeruginosa*	8	16	32
Ciprofloxacin	*Pseudomonas aeruginosa*	0.5	1	2
Gentamicin	*Pseudomonas aeruginosa*	-	-	-
Polymyxin B	*Pseudomonas aeruginosa*	-	2	8
Tobramycin	*Pseudomonas aeruginosa*	1	2	4
Tobramycin	*Staphylococcus aureus*	-	-	-
Azithromycin	*Staphylococcus* species	2	4	8
Chloramphenicol	*Staphylococcus* species	8	16	32
Ciprofloxacin	*Staphylococcus* species	1	2	4
Doxycycline	*Staphylococcus* species	4	8	16
Erythromycin	*Staphylococcus* species	0.5	1-4	8
Gentamicin	*Staphylococcus* species	4	8	16
Minocycline	*Staphylococcus* species	4	8	16
Penicillin	*Staphylococcus* species	0.125	-	0.25
Tetracycline	*Staphylococcus* species	4	8	16
Trimethoprim-Sulfamethoxazole	*Staphylococcus* species	2/38	-	4/76
Oxacillin	*Staphylococcus* species except *Staphylococcus aureus*	0.5	-	1

Cefoxitin	*Staphylococcus* species: *Staphylococcus aureus* and *Staphylococcus lugdunensis*	4	-	8
Oxacillin	*Staphylococcus* species: *Staphylococcus aureus* and *Staphylococcus lugdunensis*	2	-	4
Amikacin	*Staphylococcus* species: *Staphylococcus aureus* only	-	-	-
Ceftazidime	*Stenotrophomonas maltophilia*	8	16	32
Chloramphenicol	*Stenotrophomonas maltophilia*	8	16	32
Minocycline	*Stenotrophomonas maltophilia*	4	8	16
Trimethoprim-Sulfamethoxazole	*Stenotrophomonas maltophilia*	2/38	-	4/76
Amoxicillin	*Streptococcus pneumoniae*	2	4	8
Amoxicillin-Clavulanate	*Streptococcus pneumoniae*	2/1	4/2	8/4
Azithromycin	*Streptococcus pneumoniae*	0.5	1	2
Cefpodoxime	*Streptococcus pneumoniae*	0.5	1	2
Chloramphenicol	*Streptococcus pneumoniae*	4	-	8
Ciprofloxacin	*Streptococcus pneumoniae*	-	-	-
Doxycycline	*Streptococcus pneumoniae*	0.25	0.5	1
Erythromycin	*Streptococcus pneumoniae*	0.25	0.5	1
Tetracycline	*Streptococcus pneumoniae*	1	2	4
Trimethoprim-Sulfamethoxazole	*Streptococcus pneumoniae*	0.5/9.5	1/19-2/38	4/76
Ampicillin	*Streptococcus* viridans group	0.25	0.5-4	8
Azithromycin	*Streptococcus* viridans group	0.5	1	2
Cefotaxime	*Streptococcus* viridans group	1	2	4
Chloramphenicol	*Streptococcus* viridans group	4	8	16
Erythromycin	*Streptococcus* viridans group	0.25	0.5	1
Penicillin	*Streptococcus* viridans group	0.125	0.25-2	4
Tetracycline	*Streptococcus* viridans group	2	4	8
